# Expression, Purification, and Characterization of a Sucrose Nonfermenting 1-Related Protein Kinases 2 of* Arabidopsis thaliana* in* E. coli*-Based Cell-Free System

**DOI:** 10.1155/2016/9469356

**Published:** 2016-11-24

**Authors:** Xu Zhang, Shuangxi Zhang, Jun Wang, Jing Zi, Jianhui Wang, Shaolin Chen, Yi Wan

**Affiliations:** ^1^Microbiology Institute of Shaanxi, Shaanxi Academy of Sciences, 76 Xiying Road, Xi'an, Shaanxi 710043, China; ^2^Biomass Energy Center for Arid and Semiarid Lands, College of Life Sciences, Northwest A&F University, 3 Taicheng Road, Yangling, Shaanxi 712100, China

## Abstract

The plant-specific sucrose nonfermenting 1-related protein kinase 2 (SnRK2) family is considered an important regulator of plant responses to abiotic stresses such as drought, cold, salinity, and nutrition deficiency. However, little information is available on how SnRK2s regulate sulfur deprivation responses in Arabidopsis. Large-scale production of SnRK2 kinases* in vitro* can help to elucidate the biochemical properties and physiological functions of this protein family. However, heterogenous expression of SnRK2s usually leads to inactive proteins. In this study, we expressed a recombinant Arabidopsis SnRK2.1 in a modified* E. coli *cell-free system, which combined two kinds of extracts allowing for a convenient and affordable protein preparation. The recombinant SnRK2.1 was produced in large-scale and the autophosphorylation activity of purified SnRK2.1 was characterized, allowing for further biochemical and substrate binding analysis in sulfur signaling. The application of this improved* E. coli *cell-free system provides us a promising and convenient platform to enhance expression of the target proteins economically.

## 1. Introduction

Environmental stresses such as drought, cold, salinity, and nutrition deficiency lead to an imbalance in ionic homeostasis, oxidative damage, and growth inhibition in plants. Understanding how plants respond to these abiotic stresses is critical to improve their resistance. The sucrose nonfermenting 1-related protein kinases (SnRKs) are widely distributed in plants and act as key switches in growth and metabolic responses to cellular stress. SnRK2 family members are plant-specific kinases and important regulators in stress signaling [1]. A direct relationship has been established between abscisic acid (ABA) signaling and SnRK2 members recently [2, 3]. ABA-triggered phosphorylation of the transcription factor was successfully reconstituted* in vitro*. With ABA, the ABA-bound receptor inactivated PP2C, which caused the phosphorylation of SnRK2 kinases (SnRK2.2/2D, 2.3/2I, and 2.6/2E/OST1), allowing them to activate ABA-responsive proteins by protein phosphorylation. Besides ABA signaling, the SnRK2 subfamily also plays an important role in osmotic and drought stress signaling, as well as nutrition deprivation responses [4–6]. As a model plant, ten SnRK2-type kinases (SnRK2.1-SnRK2.10) have been identified in* Arabidopsis thaliana, *and the SnRK2s were thought to play different functional roles in this plant [7]. Sulfur deprivation, as a nutrition limitation, may result in reduced quality and yield of seeds and stunted plant growth. Sulfur deprivation induces the transcription of SnRK2.1, SnRK2.3, SnRK2.4, and SnRK2.6 in* A. thaliana*, but only one of them, SnRK2.3, has been analyzed in detail by gene depression [8]. The induction of the sulphate transporter Sultr2.2 under sulfur deprivation was disrupted in the Arabidopsis* snrk2.3* mutant, while no other sulfur starvation responses were present. Compared with SnRK2 kinases in* Chlamydomonas*, which have an important role in the control of S-starvation responses [6], Arabidopsis SnRK2.3 displays a comparatively moderate role. It indicated that other members of the Arabidopsis SnRK2 family might be able to fulfill the function of SnRK2.3 in its absence. However, the molecular targets of SnRK2 kinases, their interactions with related proteins, and the modulating mechanism of their signaling cascade remain unclear.

In order to solve these problems, large-scale production of SnRK2 kinases* in vitro* could help to elucidate their biochemical properties and physiological functions, especially in protein-protein interactions studies. To date, the preparation of SnRK2s focused on immunoprecipitation from plants and expression in* E. coli*. Production of SnRK2s in* E. coli* allows for a simple purification process and kinase activity studies independently of other coimmunoprecipitated plant proteins. SnRK2.2, SnRK2.3, and SnRK2.6 have been successfully prepared from* E. coli* [9, 10]. However, there are still problems in the production of SnRK2 kinases in* E. coli*. Previous studies indicate that* E. coli *expression of recombinant SnRKs from different plant species, including tobacco, wheat, or Arabidopsis, does not yield active proteins, likely due to the need for activation by a specific signaling cascade in plants [11–13].

The up-to-date cell-free (CF) expression system has attracted a lot of attention as an alternative tool for the production of recombinant proteins. Compared with the traditional cell culture methods, requiring a few days to prepare purified proteins, CF protein synthesis and purification only need hours. More importantly, CF system bypasses the limits imposed by the fitness of the organism and the open nature of CF systems makes their environment different from living cell systems. The discrepancy between CF and living systems in the environment might give CF systems an edge in offering the necessary components for producing active kinases, facilitating their preparation. Some kinases, along with various protein phosphatases, have been successfully prepared in CF systems, such as receptor tyrosine kinases and mitogen-activated ERK activating kinase 1 [14–16]. However, the high cost of CF systems makes it difficult to use them for large-scale protein production [17]. Here, we successfully expressed and purified Arabidopsis SnRK2.1 in large-scale (up to milligram quantities) in a modified* E. coli* CF system. And the functionality of the purified SnRK2.1 kinase was confirmed with an* in vitro* autophosphorylation assay. This may represent a starting point for further biochemical and substrate binding analysis and, thus, for a better understanding of the signaling cascade involved in S-deprivation or other abiotic stress responses.

## 2. Materials and Methods

### 2.1. Construction of the SnRK2.1 Expression Vector

The cDNA encoding for SnRK2.1 was provided by Professor Chen from College of Life Sciences, Northwest A&F University, China. The cDNA was amplified by PCR using LA Taq DNA polymerase (Takara, Dalian, China) and primer pairs as follows: 5′-CCGCCATGGACAAGTATGACGTTG-3′ and 5′-CGGGATCC *TTA*AGCTTTGTCAGACTCT-3′. Restriction sites (*Nco*I and* Bam*HI) are underlined and the stop codon TAA introduced is italicized. The PCR product was digested with* Nco*I and* Bam*HI restriction enzymes (Takara, Dalian, China) and ligated into the identically digested cell-free expression vector pIVEX2.4c (Roche Applied Science, Mannheim, Germany), carrying a His6-tag sequence at the N-terminal, by T4 DNA ligase (Takara, Dalian, China). The construction of the expression vector pIVEX2.4c-SnRK2.1, which encoded a His6-tag at the N-terminal of target protein, was confirmed by restriction enzyme digestion and the correctness of sequence was confirmed by DNA sequencing.

### 2.2. Cell-Free Protein Synthesis

**Figure 1:**
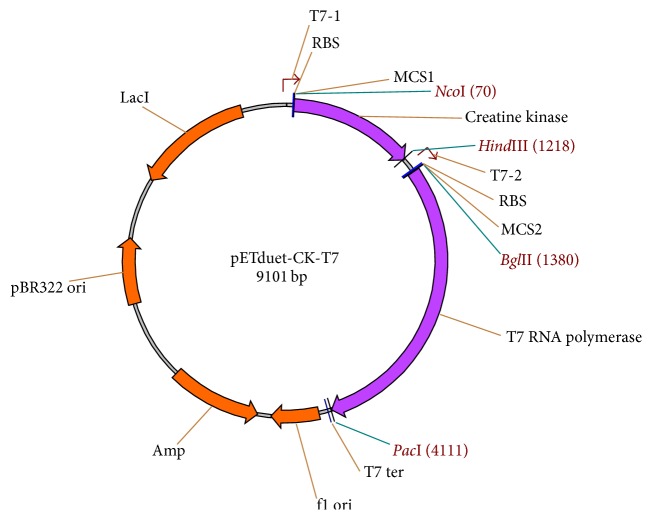
Construction of plasmid pETduet-CK-T7 for the preparation of CK-T7 extract. The creatine kinase and T7 RNA polymerase sequences were cloned into MCS1 and MCS2 of pETduet-1, respectively.

The SnRK2.1 protein was expressed in a cell-free expression system based on an* E. coli* ribosomal extract (S30 extract). S30 extract was prepared as previously reported [18] with some modifications. Briefly, in order to bypass the exogenous addition of commercial creatine kinase (CK) and T7 RNA polymerase (T7 RNAP) into the cell-free reaction mixtures, two types of S30 extracts (CK-T7 extract and BL21 extract) were prepared. The CK-T7 extract was prepared from the* E. coli* strain BL21 Star (DE3) (Life Technologies) harboring a CK and T7 RNAP expression vector pETDuet-CK-T7 (Figure 1). CK and T7 RNAP were expressed during the cultivation of* E. coli* via IPTG induction (1.0 mM) at 0.5 OD600. The cells were harvested 2 h after induction and used for preparing the CK-T7 extract. The standard BL21 S30 extract was prepared from the* E. coli* strain BL21 Star (DE3) as previously reported [18].

The standard reaction mixture consisted of the following components in a total volume of 50 *μ*L: 55 mM Hepes/KOH, pH 7.5, 1.2 mM ATP, 0.85 mM each of GTP, UTP, and CTP, 1.7 mM dithiothreitol, 200 mM K-glutamate, 0.17 mg/mL* E. coli* total tRNA, 34 mg/mL l-5-formyl-5,6,7,8-tetrahydrofolic acid (folinic acid), 0.65 mM cAMP, 2 mM each of amino acids, 80 mM creatine phosphate, 28 mM ammonium acetate, 11 mM magnesium acetate, 21 *μ*L BL21 extract, 3 *μ*L CK-T7 extract, and 10 *μ*g/mL DNA template pIVEX2.4c-SnRK2.1. The above complicated components were merged into five parts as follows: energy mix, amino acid mix, BL21 extract, CK-T7 extract, and vector DNA, to make the cell-free protein synthesis easy and convenient. For optimum productivity of target protein in the cell-free reaction mixture, a serials ratio of BL21 extract and CK-T7 extract were screened.

The pIVEX2.4c-SnRK2.1 plasmid was extracted from overnight cultures using a QIAprep Spin Miniprep Kit (Qiagen, Hilden, Germany). The reaction was carried out at 30°C with a stirring speed of 400 rpm for 3 h. Then the mixture was centrifuged at 10,000 ×g for 5 min to separate the soluble fraction and insoluble pellet. The cell-free synthesized protein was analyzed by 12% SDS-PAGE and western-blotting analysis.

### 2.3. SDS-PAGE and Western-Blotting Analysis

The size of the cell-free synthesized protein was analyzed using 12% SDS-PAGE following the standard SDS-PAGE method and stained by Coomassie brilliant blue R250. For western-blotting analysis, the gel was transferred to Nitrocellulose Membrane (Pall, New York, USA). The membrane was blocked with 5% nonfat milk and then blotted with mouse anti-his monoclonal antibody (Novagen, Madison, WI, USA) followed by HRP-conjugated goat anti-mouse IgG (Santa Cruz Biotechnology Corporation, Santa Cruz, CA, USA). DAB Color Development Kit for HRP (Beyotime Institute of Biotechnology, Haimen, China) was used to visualize the immunoreactive proteins. The sample without plasmid addition was loaded as negative control, and a recombinant protein with His6-tag (GFP) with known concentration (200 *μ*g/mL) was loaded as positive reference. The expression level of target protein was quantified by Quantity One (Bio-Red, Hercules, CA, USA).

### 2.4. Purification of Soluble SnRK2.1

Recombinant SnRK2.1 was expressed in large-scale for further purification. Each reaction was performed in a 10 mL centrifugal tube with a reaction volume of 1 mL. The soluble and insoluble fractions expressed in the CF system were separated by centrifugation. The soluble SnRK2.1 was purified in one step by immobilized metal-chelated affinity chromatography (IMAC). Briefly, 1 mL of soluble SnRK2.1 was mixed with 300 *μ*L Ni^+^ loaded NTA resin slurry (GE Healthcare, Pittsburgh, US) that had been equilibrated by column buffer A (20 mM Tris-HCl, pH 8.0, 500 mM NaCl). This solution was then diluted 7-fold with column buffer A and incubated for 2 h with gently shaking. The loaded column was washed with ten column volumes of washing buffer A (20 mM Tris-HCl, pH 8.0, 500 mM NaCl, and 20 mM imidazole), and the target SnRK2.1 was then eluted with elution buffer A (20 mM Tris-HCl, pH 8.0, 500 mM NaCl, and 300 mM imidazole). Imidazole and salts were removed by using a PD-10 desalting column (GE Healthcare, Pittsburgh, US).

### 2.5. Purification of Insoluble SnRK2.1

The insoluble fraction of SnRK2.1 expressed in our CF system was purified by extraction with 8 M urea followed by IMAC. The cell-free pellet was resuspended by column buffer B (20 mM Tris-HCl, pH 8.0, 8 M urea) and then denatured overnight. The suspension was centrifuged at 10,000 ×g for 10 min to remove any remaining insoluble matter and collect the supernatant. The corresponding supernatant was mixed with 300 *μ*L Ni^+^ loaded NTA resin slurry and incubated for 2 h with shaking gently. The loaded column was washed with ten column volumes of washing buffer B (20 mM Tris-HCl, pH 8.0, 20 mM imidazole, and 8 M urea), and the elution buffer B (20 mM Tris-HCl, pH 8.0, 300 mM imidazole, and 8 M urea) was applied to elute the target SnRK2.1.

The elution fraction was refolded by stepwise dialysis against a 50-fold excess of refolding buffer (20 mM Tris-HCl, pH 8.0) containing 4 M, 2 M, 1 M, or 0 M urea. Each dialysis step lasted for at least 2 h. The solution was dialyzed in nonurea buffer again and then centrifuged at 12,000 rpm for 15 min at 4°C. The protein samples were analyzed by SDS-PAGE.

### 2.6. Kinase Assay of Purified SnRK2.1

For kinase assay of the SnRK2.1, autophosphorylation kinetics of SnRK2.1 purified from soluble and insoluble fraction were measured by using the EnzyChrom™ Kinase Assay Kit (BioAssay Systems, Hayward, US), respectively. Kinase activity was measured by the quantification of ADP, which was enzymatically converted to ATP and pyruvate, using a fluorimetric (530 nm/590 nm) assay method. 0.5 *μ*g purified SnRK2.1 was incubated in 32 *μ*L reaction buffer provided by the kit with 150 *μ*M ATP at 20°C for the indicated time in each kinase assay (40 *μ*L reaction volume). As the blank control, the reaction buffer mixed with 150 *μ*M ATP without enzyme was also incubated for the indicated time. After incubation with the working agent for 10 min, the fluorescence intensity was read at *λ*exc = 530 nm and *λ*em = 590 nm. The standard curve was performed as indicated by the kit. The kinase activity was calculated as the instruction of the EnzyChrom Kinase Assay Kit. Specifically, Kinase Activity = (Δ*F*_SAMPLE_/(Slope · *t*)) × (40 *μ*L/Vol (*μ*L)) (U/L), where Δ*F*_SAMPLE_ = (fluorescence intensity of sample well − fluorescence of the blank well); slope is the slope of the ADP standard curve. *t* is the kinase reaction time (5 min was adopted here). Vol is the volume (*μ*L) of kinase added to the 40 *μ*L reaction.

## 3. Results and Discussions

### 3.1. Expression of SnRK2.1 in a Cell-Free System

**Figure 2:**
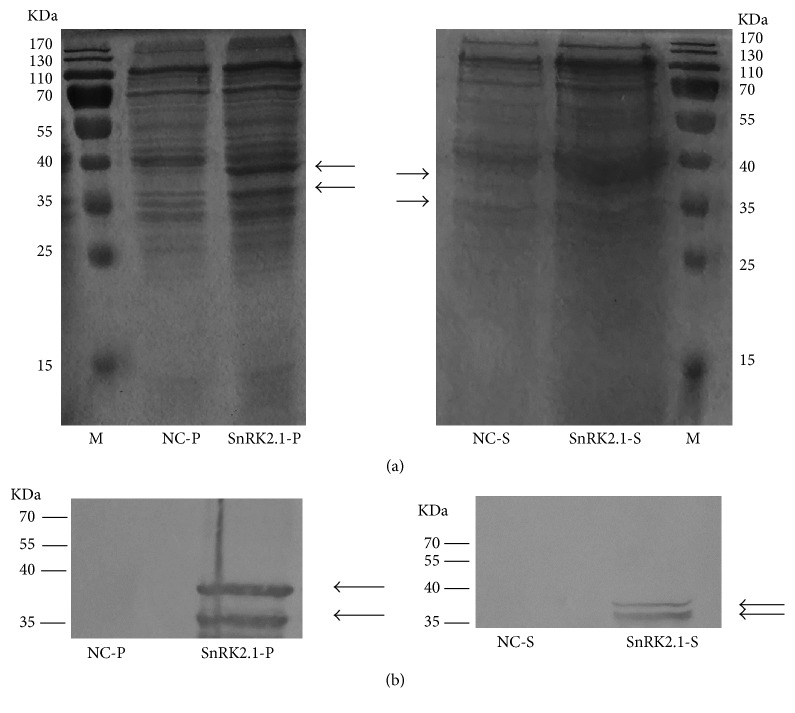
SDS-PAGE and western-blotting analysis of insoluble and soluble recombinant SnRK2.1. The reaction was performed in a volume of 50 *μ*L and the soluble fraction and insoluble pellet were separated by centrifugation. 5 *μ*L of the precipitate (P) and 5 *μ*L of the supernatant (S) were analyzed by SDS-PAGE (a), while 1 *μ*L of the precipitate and 1 *μ*L of the supernatant were analyzed by western-blotting (b). The arrow marks indicate the molecular mass of the recombinant SnRK2.1. M, maker; NC-P, precipitate of negative control without plasmid; SnRK2.1-P, precipitate of recombinant SnRK2.1; NC-S, supernatant of negative control without plasmid; SnRK2.1-S, supernatant of recombinant SnRK2.1.

The expression of recombinant SnRK2.1 in* E. coli *using pCold plasmid and BL21 strain was not detected in our preliminary experiments (data not shown). The DNA fragment (1062 bps) encoding for Arabidopsis SnRK2.1 was inserted into a pIVEX2.4c vector at the* Nco*I and* Bam*HI sites, resulting in the recombinant plasmid pIVEX2.4c-SnRK2.1, which was used for the cell-free protein synthesis reactions. Small-scale expression was performed in a reaction volume of 50 *μ*L, and the soluble fraction and insoluble pellet were separated by centrifugation. We confirmed the successful expression of our target protein in both soluble and insoluble forms by SDS-PAGE and western-blotting analysis (Figures 2(a) and 2(b)). The presence of recombinant protein matching the excepted molecular mass of 40 kDa encoded by a 1059 bp ORF was detected. SnRK2 family members are of about 40 kDa in size and have a characteristic aspartic/glutamic acid-rich patch in their C-terminal domain [19]. The minor bands observed in SDS-PAGE or western-blotting analysis were probably due to protein degradation or premature translation termination, considering the characteristic C-terminal structure of SnRK2.1. The quantity of insoluble SnRK2.1 (about 250 *μ*g/mL) was higher than that of the soluble SnRK2.1 (about 60 *μ*g/mL) (Figure 2(b)). Some detergents are able to increase the expression level of soluble membrane proteins in CF system [20]. Thus, in order to enhance the production of soluble SnRK2.1, we tried different detergents, such as polyoxyethylene-(20)-cetyl-ether (Brij58), TritonX-100, and Tween 80. However, we observed no obvious effects (data not shown). Therefore, the detergent strategy might not be suitable for the characteristic structure of SnRK2s.

### 3.2. Expression of SnRK2.1 by Using Optimized CF System

**Figure 3:**
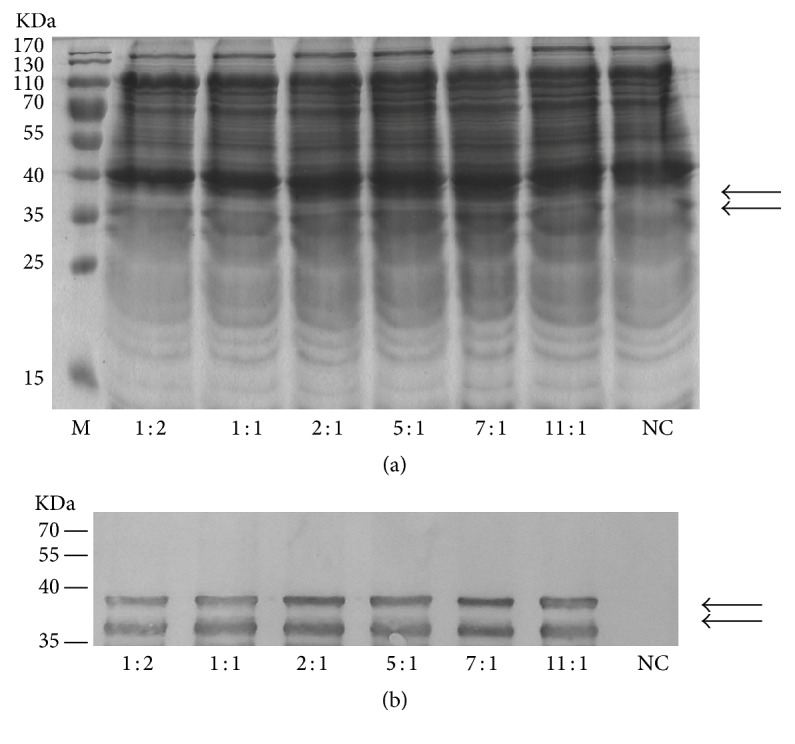
The influence of different ratio of BL21 extract and CK-T7 extract on expression of SnRK2.1 in cell-free system. The recombinant protein was expressed with 8 *μ*L BL21/16 *μ*L CK-T7 extract (1 : 2), 12 *μ*L BL21/12 *μ*L CK-T7 extract (1 : 1), 16 *μ*L BL21/8 *μ*L CK-T7 extract (2 : 1), 20 *μ*L BL21/4 *μ*L CK-T7 extract (5 : 1), 21 *μ*L BL21/3 *μ*L CK-T7 extract (7 : 1), and 22 *μ*L BL21/2 *μ*L CK-T7 extract (11 : 1), respectively. The reactions were terminated by mixing with loading buffer and then analyzed by SDS-PAGE (a) and western-blotting analysis (b). M, maker; NC, negative control without plasmid addition. The arrow marks indicate the molecular mass of the recombinant SnRK2.1.

The emerging cell-free system is a powerful tool for production of cytotoxic, unstable, or insoluble proteins. Nevertheless, their high cost limits their use for laboratory research purposes. Many researchers have tried to improve protein productivity and cost-efficiency of* E. coli*-based systems in the last decades [21, 22]. It was reported that purification of T7 RNAP was time-consuming and unnecessary, and combining T7 RNAP and extract into a single reagent by IPTG induction of T7 RNAP expression during the exponential growth phase simplified the purification protocol for T7 RNAP [23, 24]. Here, we further improved this protocol by using two different cell extracts. Both the creatine kinase and T7 RNAP genes were cloned into one single plasmid and the enzymes were induced simultaneously, followed by preparation of CK-T7 extract. The CK-T7 extract provided the necessary enzymes to reduce the cost of our CF systems, while the standard BL21 extract guaranteed the productivity. We screened the ratio of the two extracts to produce recombinant SnRK2.1 economically. The CK-T7 lysate was less productive than the standard lysate, and the optimum ratio of BL21 and CK-T7 extracts for the preparation of SnRK2.1 was 7 : 1 (Figures 3(a) and 3(b)). Our optimized CF protocol eliminated the addition of T7 RNAP and CK, allowing for simplified experimental applications and reduced costs compared with previous systems; thus, it can be applied as a powerful factory to produce target proteins. There are instances where it would be advantageous to use extracts produced from strains other than BL21 (DE3) for various applications, such as formation of disulfide bonds and incorporation of nonnatural amino acids. This method could be further improved by using other extracts for specific reasons together with the CK-T7 extract to simplify the procedure and reduce the costs.

### 3.3. Large-Scale Expression of SnRK2.1 and Subsequent Purification

**Figure 4:**
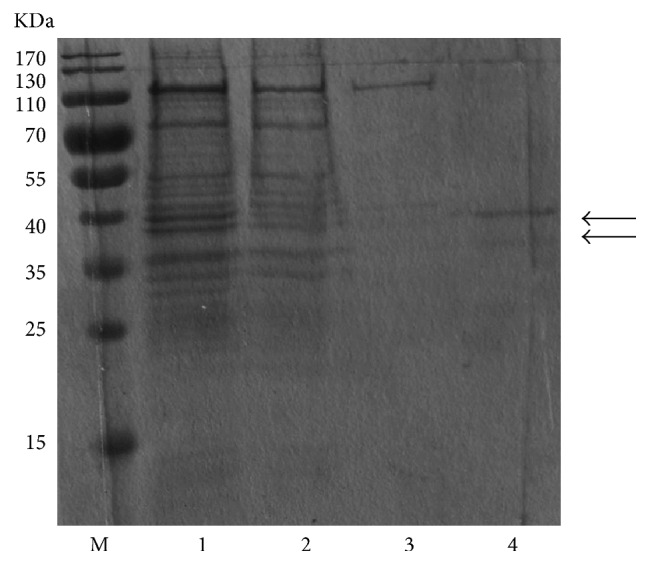
Purification of the soluble recombinant SnRK2.1 expressed in cell-free system. Reaction mixture was centrifuged to separate soluble and insoluble fractions. Soluble SnRK2.1 was diluted with column buffer A and directly purified using Ni-NTA column. M, maker; Lane 1, diluted supernatant sample; Lane 2, flow through; Lane 3, washing with 20 mM imidazole; Lane 4, elution with 300 mM imidazole.

Based on the result of our initial screen of the extract ratio, 7 : 1 (BL21 : CK-T7) was used for further experiments aimed at achieving large-scale production of SnRK2.1. Since the expression level of soluble SnRK2.1 was comparatively low, we aimed to purify both soluble and insoluble SnRK2.1 separately to investigate their biological activities. Recombinant SnRK2.1 protein was expressed as a His6-tag fusion protein. We purified soluble recombinant SnRK2.1 using nickel resin affinity chromatography. As shown in Figure 4, fractions eluted from the affinity column with 300 mM imidazole mainly contained recombinant SnRK2.1 with a size of around 40 kDa, which closely correlates with the predicted protein molecular mass. The additional faint protein bands at lower molecular mass were consistent with our western-blotting analysis of expression (Figure 2(b)); therefore, we speculated that this was the truncated target protein. The yield of the purified soluble recombinant protein varied from 20 to 30 mg/L of the reaction mixture.

**Figure 5:**
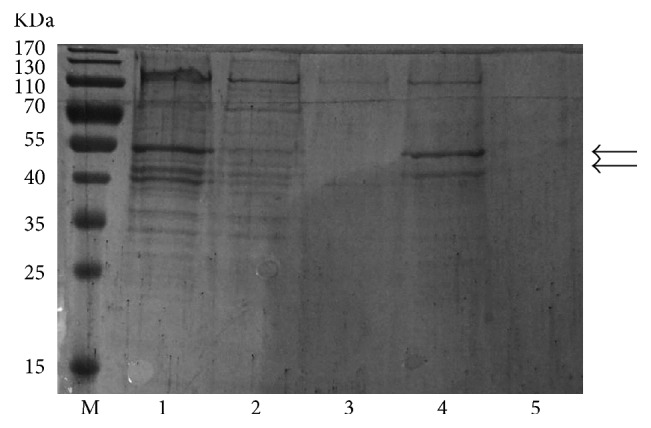
Purification of the insoluble recombinant SnRK2.1 expressed in cell-free system. Insoluble SnRK2.1 was resuspended with column buffer B containing 8 M urea and then purified using Ni-NTA column. M, maker; Lane 1, resuspension with 8 M urea; Lane 2, flow through; Lane 3, washing with 20 mM imidazole; Lane 4, elution with 300 mM imidazole; Lane 5, pellet could not be dissolved by urea.

We also performed the purification of the insoluble recombinant SnRK2.1 using IMAC, adding 8 M urea for the denaturation and resuspension steps. Most precipitated protein was dissolved in the 8 M urea buffer and no protein was left in the pellet (Figure 5). The subsequent purification steps were all carried out using buffers with 8 M urea. The denatured protein was eluted with 300 mM imidazole and purified effectively. The truncated protein was also observed in the eluate. Functional proteins must be folded correctly. Thus, the eluted protein was refolded by using a stepwise dialysis to remove urea and imidazole. The final yield of the recombinant SnRK2.1 purified from the precipitate of CF expression system varied from 90 to 100 mg/L of the reaction mixture.

It has been reported that some recombinant SnRKs expressed in* E. coli* were inactive, probably due to the lack of specific signaling cascades activating them [11–13]. Moreover, the use of different tags might affect the activity of the prepared proteins. For example, a short 6xHis tag fused at the N terminus does not allow the purification of native recombinant protein SnRK2.6, while 10xHis-tagged proteins can be successfully purified [9]. However, the exact nature of the factors influencing the activity of purified SnRKs remains unknown. In this study, we purified low molecular mass proteins together with our target protein in both soluble and insoluble SnRK2.1 fractions, which implied the presence of a truncated protein. The C-terminal regulatory domain of SRK2E/OST1/SnRK2.6 is required for its activation by both ABA and osmotic stress in Arabidopsis [2]. At the same time, a carboxy-terminally truncated SnRK2.6 lacking of the amino acids 280–362 does not interact with the type 2C protein phosphatase ABI1 [3]. Considering the complicated C-terminal structure of SnRK2.1, the truncated protein in this study might be a consequence of protein translation termination or protease degradation. We also analyzed a His6-tag C-terminal fusion. However, we found that the molecular mass of the recombinant protein was about 55 kDa in the SDS-PAGE and western-blotting analysis (data not shown). Considering the acidity of the amino acids in the C-terminal domain of SnRK2.1, the fusion of His6-tag at N-terminal could avoid its interference with the structure or function of SnRK2.1 as far as possible. We obtained a purity of approximately 90% and yield of 20–30 mg/L for soluble SnRK2.1 and a purity of approximately 70% and yield of 90–100 mg/L for insoluble SnRK2.1, which was sufficient for measuring protein activity.

### 3.4. Kinase Activity of Purified SnRK2.1

**Figure 6:**
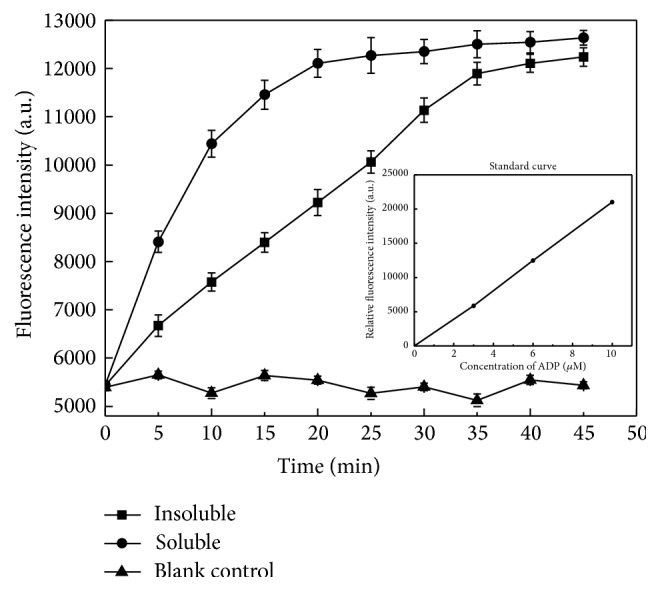
Autophosphorylation kinetics of recombinant SnRK2.1. The autophosphorylation of recombinant SnRK2.1 purified from soluble (closed circles) and insoluble (closed squares) fractions were assayed separately. Closed triangles indicate the blank control. Error bars represent the standard deviations calculated from three individual measurements. The inset is ADP standard curve performed as instruction of the EnzyChrom Kinase Assay Kit.

SnRK2 subfamily members have a kinase domain, ATP binding domain, serine/threonine active site, and four N-fourteen sites [25]. SnRK2s can phosphorylate a number of proteins to regulate their activity while also showing autophosphorylation activity [10]. We performed an autophosphorylation kinetic study using SnRK2.1 purified from soluble and insoluble fractions expressed in our* E. coli* cell-free system. The fluorescence intensity of the blank control remained almost unchanged over time (Figure 6). On the other hand, the fluorescence intensity of both the reaction mixture containing soluble or insoluble SnRK2.1 increased over time and showed a tendency to saturation, which indicated autophosphorylation activity. In addition, the autophosphorylation of SnRK2.1 purified from the soluble fraction saturated after 20 min, while that of the SnRK2.1 purified from the insoluble fraction saturated after 35 min. We calculated the kinase activity of SnRK2.1 from soluble and insoluble fraction as 1.63 U/L and 0.68 U/L, respectively, and their specific activities as 22.9 U/g and 9.6 U/g. These results might be caused by the differences in the purity of the proteins, considering that the purity of SnRK2.1 from the soluble fraction was higher than that from the insoluble fraction. On the other hand, incomplete refolding of SnRK2.1 may also account for the discrepancy in the autophosphorylation activities.

Arabidopsis SnRK2s carries several phosphorylation sites and phosphorylation is essential for their activation. Depending on the phosphorylation state of each site, these proteins may display a specific phosphorylation pattern corresponding to a particular signaling role. There are different signaling pathways that can activate SnRK2s. For example, activation of SnRK2.2, SnRK2.3, and SnRK2.6 likely requires both* de novo* phosphorylation and modification of the already phosphorylated sites, whereas other SnRK2s are only activated by* de novo* phosphorylation [26]. A small modification of the phosphorylation level can greatly modify the activation level of the kinases. In this context, we plan on identifying the phosphorylation sites of SnRK2.1 and investigate its phosphorylation state during S-deprivation, in order to study its role in S-deprivation responses. Also, large-scale production of SnRK2.1 in our cell-free system makes it possible to investigate the substrates of this protein in various signaling pathways and study protein-protein interactions. Identifying the molecular targets of SnRK2 kinases will provide a starting point to understand how these proteins modulate plant growth and development.

## 4. Conclusions

SnRK2s are important regulators of abiotic stress responses. In this study, we performed a large-scale production of Arabidopsis SnRK2.1 in a modified* E. coli* CF system using two different S30 cell extracts. The recombinant protein was successfully purified from both the soluble and insoluble fractions of CF reaction and its autophosphorylation activity was verified. Thus, the system described here is a powerful tool for SnRK2 production and will be useful for the study of phosphorylation patterns, substrate identifications, protein-protein interactions, and other biochemical properties, which in turn would lead to a better understanding of the signaling cascade involved in S-starvation and other abiotic stress responses. Besides, our improved CF system provides us a promising and convenient factory for large-scale production of other recombinant proteins.
